# Determining gene specificity from multivariate single-cell RNA sequencing data

**DOI:** 10.1101/2025.11.21.689845

**Published:** 2025-11-23

**Authors:** Nikhila P. Swarna, A. Sina Booeshaghi, Elisabeth Rebboah, M. Grace Gordon, Pooja Kathail, Taibo Li, Marcus Alvarez, Chun Jimmie Ye, Barbara Wold, Ali Mortazavi, Lior Pachter

**Affiliations:** 1 Division of Biology and Biological Engineering, California Institute of Technology, Pasadena, CA, USA; 2 Department of Computing and Mathematical Sciences, California Institute of Technology, Pasadena, CA, USA; 3 Department of Bioengineering, University of California at Berkeley, Berkeley, CA, USA; 4 Department of Developmental and Cell Biology, University of California at Irvine, Irvine, CA, USA; 5 Center for Complex Biological Systems, University of California Irvine, Irvine, CA, USA; 6 Biological and Medical Informatics Graduate Program, University of California, San Francisco, CA, USA; 7 Division of Rheumatology, Department of Medicine, University of California, San Francisco, CA, USA; 8 Institute for Human Genetics, University of California, San Francisco, CA, USA; 9 Department of Bioengineering and Therapeutic Sciences, University of California, San Francisco, CA, USA; 10 Center for Computational Biology, University of California, Berkeley, Berkeley, CA, USA; 11 Department of Biomedical Engineering, Johns Hopkins University, Baltimore, MD, USA; 12 Gladstone-UCSF Institute of Genomic Immunology, San Francisco, CA, USA; 13 Department of Epidemiology and Biostatistics, University of California, San Francisco, San Francisco, CA, USA; 14 Bakar Computational Health Sciences Institute, University of California, San Francisco, San Francisco, CA, USA; 15 Parker Institute for Cancer Immunotherapy, University of California, San Francisco, San Francisco, CA, USA; 16 Arc Institute, Palo Alto, CA, USA

## Abstract

An important application of single-cell genomics experiments is to identify genes specific to biological categories or experimental conditions. Although numerous approaches have been proposed to identify such genes, we consider an axiomatic approach based on defining properties that a specificity measure should have. This leads us to develop ember (Entropy Metrics for Biological ExploRation), which we show is the only method satisfying four key desired properties for a specificity measure. Applying ember to eight tissues from eight founder mouse strains, we find that gene specificity is often unintuitive: canonical markers can be supplanted, housekeeping genes are context-dependent, and mouse strain can drive unexpected cell type switching. Unsupervised learning on entropy metrics uncovers shared genes specialized to male gonads and kidney, as well as genes specific to non-consecutive developmental stages in the kidney. To facilitate further exploration of gene specificity in mice, we have also developed a comprehensive specificity database, along with a web interface and API. Extending ember to a human PBMC dataset collected from 255 diverse individuals, we find that variation in PBMCs is largely localized to classical monocytes. We also find genes with unique specificity by sex, age and ancestral background. Together, these applications establish ember as a powerful tool and provide a roadmap for elucidating the impact of human genetic variation using the murine model.

## Introduction

With the advent of combinatorial barcoding and split-pool technologies in single-cell RNA sequencing (scRNA-seq), experiments have become increasingly high-dimensional (1, 2). Modern experiments not only assay tens of thousands of genes, but can also accommodate replicates across multiple biological variables: sex, tissue, age, genotype, and cell states (3–8). Although this expansion in experimental size paired with an increased depth of sequencing allows unprecedented resolution of cellular and molecular biology, there is a lack of tools to appropriately dissect datasets and extract meaningful insights (9–11).

One challenge in analyzing such multivariate data is the identification of genes that are highly specific to a given variable. Several measures of specificity have been proposed to address this challenge. For example, Tau, or the tissue specificity, index (TSI) summarizes gene specificity into a single number bounded between 0 and 1, with 0 indicating broad expression and 1 indicating perfect specificity (12, 13). The preferential expression measure (PEM) assigns positive or negative values to indicate over- or under-expression in a given tissue (14, 15), producing tissue-by-tissue scores that can then be aggregated. The Gini index, originally developed in economics to quantify inequality, has also been adapted to describe the distributional unevenness of gene expression across tissues (16, 17). Specificity indices have also been studied empirically (18). While such studies inform discrepancies between methods, they rely on the number of genes identified as specific to assess the quality of a method, ignoring its intrinsic properties (or lack of properties). In particular, the issue of specificity in the context of multivariate data seems to have been largely overlooked. Moreover, a major limitation of existing specificity measures in multivariate contexts is their lack of decomposability: the specificity does not decompose hierarchically in a coherent manner, and depends on the order in which variables are partitioned, preventing consistent application of indices such as TSI across multiple dimensions. Conversely, generalized linear model (GLM) frameworks such as DESeq2 (19) and EdgeR (20), offer decomposability but rely on parametric assumptions and can scale poorly.

Ideally, a specificity measure should satisfy several natural properties. It should be bounded between 0 and 1 indicating least and most specificity respectively, be insensitive to the ordering of categories, vary smoothly with the data, and behave coherently under hierarchical refinements in multimodal data (Supplementary Note). We show that an entropy-based approach, grounded in an axiomatic information theory framework (21, 22), provides a natural foundation for such a measure. This approach has been considered before (23), however, its reliance on the Kullback–Leibler divergence breaks key desirable additivity properties. In addition to these natural properties, any programmatic implementation should leverage single-cell resolution, efficiently scale to high-dimensional data and be robust to biological replicates for ease of use in practice.

We propose a set of three biologically informative entropy metrics: Ψ (Psi): fraction of information explained by a partition, $\psi_{\text{block}}$ (psi_block): specificity to a block, and ζ (Zeta): specificity to a partition. Detailed derivations and interpretations are provided in the Methods section.

Together with novel visualization strategies, these metrics provide a principled and interpretable toolkit for studying biological specificity in high-dimensional single-cell data. In fact, we show that our specificity measure is the only one that satisfies four basic requirements that a specificity measure should have (Supplementary Note). We implemented our approach in a Python package called ember: Entropy Metrics for Biological ExploRation. In the following sections, we apply ember to three multivariate single-cell datasets: snRNAseq of 8 tissues from 8 individuals for each of the 8 founder mouse strains (8cube founder data (6)), scRNAseq of murine kidney at 6 developmental time points (developmental kidney data (7)), and scRNAseq of human PBMCs from 255 diverse individuals (human PBMC data), demonstrating the framework’s ability to uncover novel condition-specific genes as well as larger-scale biological patterns.

## Results

### Gene specificity is unintuitive and hierarchical additivity allows for novel gene discovery.

#### Strain-specific genes.

**Fig. 1.**
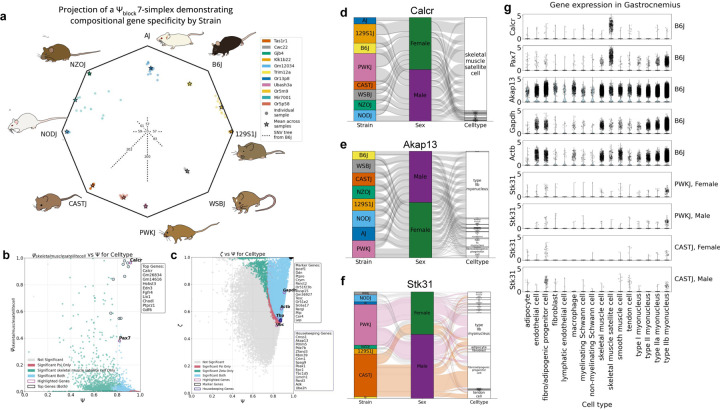
Visualizing gene specificity and finding highly specific and non-specific genes **a.** Octagonal plot showing gene specificity in compositional space. The 8cube founder dataset (6) was partitioned by strain to generate $\psi_{\text{block}}$ scores for each of the eight founder strains. Each gene is represented as a coordinate on a 7-simplex, where each vertex indicates perfect specificity to one strain. We project genes according to single nucleotide variant distance to B6J. Selected genes show specificity to one or two strains. Fifty balanced samples across biological replicates were used to generate $\psi_{\text{block}}$ scores. Each sample is shown as a grey circle, and the Aitchison mean of these 50 samples is shown as a star. Mouse images from Saul *et al*. 2019 (24) **b.** Scatter plot showing gene specificity to skeletal muscle satellite cells in 8cube gastrocnemius (6), partitioned by cell type. Genes with high Ψ and high $\psi_{\text{skeletalmusclesatellitecells}}$ are identified as marker genes (highlighted in black). Significance testing for both metrics is shown by color: genes significant for both are light blue and exhibit reliable specificity. Top marker genes are listed in the top-right text box. **c.** Scatter plot showing gene specificity in 8cube gastrocnemius (6), partitioned by cell type. Genes with high Ψ and high ζ are highly specific (highlighted in black), whereas those with high Ψ and low ζ are non-specific (highlighted in violet). Canonical housekeeping genes are highlighted in purple. Top cell type–specific genes are listed in the top-right text box, and top housekeeping genes are listed in the bottom-right text box. **d, e, f.** Alluvial plots depicting pseudo-bulked gene expression of *Calcr, Akap13* and *Stk31* across strains, sexes and cell types in 8cube gastrocnemius (6), generated using wompwomp (25). **g.**Violin plots of gene expression in 8cube gastrocnemius (6), with gene names on the left and strain/sex annotations on the right.

Applying ember to the 8cube founder data set (6), we identified genes that are strain-specific across all eight tissues (Fig. 1a). We identified *Cwc22*, which encodes spliceosome-associated protein 58, as a WSBEiJ (WSBJ) specific gene. *Cwc22* has been documented to have heightened expression in WSBJ mice due to a copy number expansion at the *R2d2* locus, located approximately 6 Mb downstream from its paralog *R2d1* (26) (Supp. Fig 1c). We also identified the gap junction protein encoded by *Gjb4*, as specific to NZO/HlLtJ (NZOJ) (Supp Fig 1d). It has been documented that *Gjb4* expression in diabetic NZOJ mice is enhanced by a reduced *miR-341–3p* expression (27).

We identified *Tas1r1* as a CASTEiJ (CASTJ) specific gene, with a slight bias towards females (Supp Fig 1a). *Tas1r1* encodes a member of the taste receptor family of genes, and functions as a receptor of umami taste along with another member of the same family, *Tas1r3* (28). Interestingly, it has also been observed that this wild-derived strain displays a unique oral preference to sugars, distinguishing CASTJ from other strains of mice and rats (29). When comparing transcriptomic differences between mouse strains mapped to the standard GRCm39 reference genome, we run the risk of falsely quantifying mapping artifacts as strain specificity becauase the GRCm39 assembly is derived from a C57BL/6J (B6J) background (6). To exclude the possibility that *Tas1r1*’s specificity to CASTJ mice could be the result of such a mapping artifact, we examined its coding sequence from the recent first release of the telomere-to-telomere (T2T) genomes for B6J and CASTJ (30, 31) (Supp. table 1). There is no evidence of frameshift or nonsense mutations and no alternative splicing, as the total CDS and protein length are conserved. This indicates that the *Tas1r1* gene in B6J is not a pseudogene in CASTJ. Between the strains, 19 SNPs occur in exonic regions, of which three cause amino acid substitutions that may alter protein structure. We observed a large 4396 base pair deletion in B6J compared to CASTJ in the fourth intron at the CASTJ T2T coordinates 4:153181901–153186296 (reverse strand). Large intronic deletions have been associated with over or under expression of genes (32–34), thus cluing why we may be observing this specificity of *Tas1r1* to CASTJ. The gene just upstream of *Tas1r1* on the reverse strand chromosome four, *Zbtb48*, also exhibits biased expression towards CASTJ, though not as striking as that of *Tas1r1* (Supp Fig 1b), further validating our finding.

In addition to strain-specific genes, we also observed genes that are specific to pairs of strains. *Or5m9* encodes an olfactory receptor specific to B6J and 129S1/SvImJ (129S1J). Another olfactory receptor *Or5p58* is specific to the wild-derived strains PWK/PhJ (PWKJ) and CASTJ, the most genetically distant from B6J of the eight founder strains. *Mir7001*, encodes a microRNA specific to the two diabetic mouse strains NZOJ and NOD/ShiLtJ (NODJ). Additionally, we found that gene specificity to mouse strain is variable across biological replicates. We applied a novel visualization strategy to depict $\psi_{\text{block}}$ scores for each founder mouse strain and their mean and standard deviation values in compositional space as an octagon, where proximity to a vertex represents greater specificity (Fig. 1a, detailed explanation in Methods). These results show that ember can identify known and novel genes that are specific to one or more mouse strains.

#### Cell type markers and housekeeping genes.

Our method ember can be used to find cell type markers and housekeeping genes, while taking into account all possible partitions in a multivariate dataset. We found markers for skeletal muscle satellite cells, a rare but essential stem cell population required for adult muscle repair, making up only 1% of all nuclei in the 8cube gastrocnemius dataset (6) (Fig. d). The top satellite stem cell marker to emerge from our analysis was *Calcr*, calcitonin receptor (Fig. 1b, 1d). *Calcr* maintains the muscle stem cell pool by keeping them in a dormant state and in their location via the *cAMP-PKA* pathway (35). We were surprised that *Pax7*, the canonical myogenic marker, did not appear in our list of top satellite cell markers. In our analysis, *Pax7* was significant after permutation testing by $\psi_{\text{satelitecells}}$ but not by Ψ (Fig 1b), which means that *Pax7* has some biased expression in satellite cells, but partitioning by cell type does not explain its expression pattern. Examining total expression across all gastrocnemius cell types, we found that *Pax7* also has significant expression in type IIB myoneuceli, the most populous of the gastrocnemius cell types (Fig. 1g). We validated these findings by comparing to a gastrocnemius single-nucleus dataset generated by Alexander S. Ham *et al*. 2025 (36), where we observed the same expression of *Pax7* in both satellite cells and type IIB myoneuceli (Supp. Fig. 2).

Selecting for genes partitioned by cell type with high Ψ and low ζ, we found housekeeping genes for the 8cube gastrocnemius (6) (Fig. 1c). From our analysis, *Akap13*, which encodes an A kinase receptor protein, emerged as one of the top housekeeping genes by cell type (Fig. 1e). We found that *Akap13* outperforms several of the canonical housekeeping genes such as *Gapdh, Actb, Ubc* and *Tbp* (Fig 1c, 1g). Moreover, it has been documented that *Gapdh* mRNA is highly variable across tissues and cell types (37), making it an unreliable default housekeeping gene. This result illustrates the advantage of taking a context-aware approach to housekeeping gene selection using ember.

#### Strain-driven cell type specificity.

We also uniquely find genes that switch cell type specificity based on strain. *Stk31* encodes serine threonine kinase 31 which enables protein kinase activity. Bao *et al*. 2013 report that *Stk31* is exclusively expressed in spermatogenic cells of testes in mice, their study however is limited to mice of a C57BL/6J background (38). While in the 8cube dataset we observe that *Stk31* expression is generally highest in male gonads, specifically in CASTJ we observe some expression in multiple other tissues (Supp. Fig 1e). For instance in gastrocnemius, we observe that it is selectively expressed in fibro-adipogenic progenitor cells in CASTJ mice but in type IIb myonuclei in PWKJ mice (Fig. 1g). This strain-dependent expression reveals that genetic background can drive cell type specificity, challenging the notion of universal cell type markers. This especially motivates exercising caution when drawing cross-strain and cross-species conclusions from a mouse model. To fuel effective and informed use of mouse models in biological research, we have deposited specificity and gene expression information across the 8 diverse founder strains in 8 tissues into a publicly available and programatically accessible API database and website (Data Availability). Together, these results showcase the ability of ember as a tool to evaluate the efficacy of marker and housekeeping genes to a partition while also identifying novel strain-aware cell type-specific genes.

### Unsupervised learning from entropy metrics can identify clusters of specific genes.

#### Clustering by tissue.

**Fig. 2.**
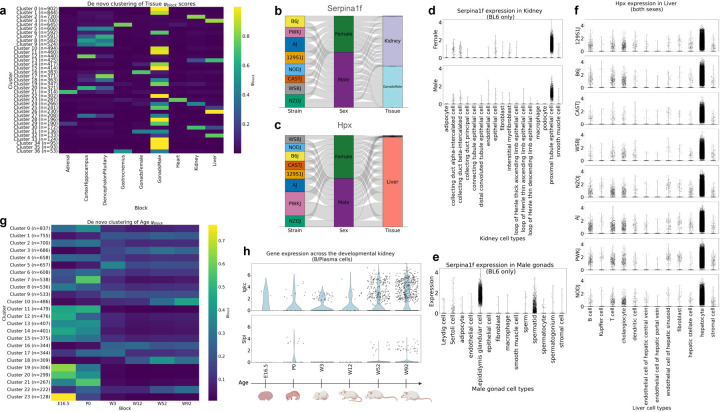
Unsupervised clustering of $\psi_{\text{block}}$ scores. **a.** Heatmap showing de novo unsupervised clustering of $\psi_{\text{block}}$ scores generated from the 8cube dataset (6), partitioned by tissue. **b, c.** Alluvial plots depicting pseudo-bulked gene expression of *Serpina1f* and *Hpx* across strains, sexes and tissues in 8cube (6), generated using wompwomp (25). **d.** Violin plots of *Serpina1f* gene expression in 8cube B6J kidney in each sex, across cell types (6). **e.** Violin plot of *Serpina1f* gene expression in 8cube B6J male gonads across cell types (6). **f.** Violin plots of *Hpx* gene expression in 8cube Liver in both sexes, across strains and cell types (6). **g.** Heatmap showing de novo unsupervised clustering of $\psi_{\text{block}}$ scores generated from the developmental kidney dataset (7), partitioned by age. **h.** Violin plots of *Igkc* and *Slpi* gene expression in the B/Plasma cells of developmental kidney in both sexes, across ages (7), mouse developmental timeline image on x-axis created in https://BioRender.com.

We performed unsupervised machine learning using the leiden clustering algorithm on the entire 8cube data, clustering on $\psi_{\text{block}}$ scores of each tissue (Fig. 2a) (6, 39). We found 4863 out of the 14181 genes with Ψ>0.45,ζ>0.45 were specific to male gonads (34%). This is in agreement with previous work in humans by Djureinovic *et al*. 2014, which confirms that male gonads are the tissue with most tissue-specific genes (40). We also found clusters of genes that are specific to pairs of tissues involving male gonads (Table 1, Supp. tables 2–8). After performing a functional enrichment analysis (41) of the 226 genes with shared specificity to kidney and male gonads, we found 68 genes are regulated by a *Twist* transcription factor. *Twist1* and *Twist2* are paralogs in the *Twist* subfamily of basic loop-helix-loop proteins, which are essential for embryogenesis and are conserved across vertebrates and invertebrates (42, 43). The role of *Twist1* has been extensively studied in the kidney and is implicated in kidney disease (44–46). However, previous research has found that Twist1 is not detected in the murine testes (47), so these genes with shared regulation by *Twist* family in both male gonads and kidney lead us to a novel line of biological questioning.

*Serpina1f*, which encodes serine peptidase inhibitor, clade A member 1f, is one of the 68 genes specific to male gonads and kidney and regulated by *Twist* (Fig 2b). *Serpina1f* has been previously identified as kidney-specific in a semiquantitative PCR analysis study of adipose tissue, muscle, heart, lung, liver and kidney (48). We see from our data that *Serpina1f* is largely localized to proximal tubule epithelial cells in both males and females (Fig 2b, 2d). In male gonads, *Serpina1f* has been localized to mouse epididymis in an RNA in-situ hybridization study (49, 50) and to sperm cell membrane in a proteomics study (51). We also observed localization of *Serpina1f* to epididymis and spermatid in male gonads (Fig 2e). These results validate that *Serpina1f* is reproducibly and distinctly observed in both kidney and male gonads.

The only tissue with a cluster of highly specific genes ($\psi_{\text{block}}>0.8$) other than male gonads was Liver (Fig 2a, Cluster 26, 230 genes). A functional enrichment analysis on this cluster of genes revealed regulation of large sets of these genes by six transcription factors: *Tcf-1, Cdx-2, Crx, Mef-2c, Sox-10* and *Cdp*. *Tcf-1* or *Hnf1a* encodes a liver-enriched transcription factor critical to several aspects of liver function (52–56). *Hpx* is one of the 26 genes that we identified as liver-specific and regulated by *Tcf-1* (Fig 2c, Supp. table 3, 8). *Hpx*, encodes hemopexin, a protein that binds heme and transports it to the liver for breakdown and iron recovery. While *Hpx* is visibly specific to liver across all 8 tissues (Fig 2c), at the strain level we noticed that *Hpx* shows a slight bias towards PWKJ mice, which are the most prone to develop hepatic inflammation of the founder strains (57). At the cell type level, we noticed localization of *Hpx* to hepatocytes across all strains (Fig 2f). These results show that we can find clusters of biologically meaningful genes with tissue specificity.

#### Clustering by age.

We used ember to understand gene specificity in a developmental kidney dataset produced by Chen *et al*. 2025. In this data, the developing kidney is captured in six stages: E16.5, P0, week 3 (W3), week 12 (W12), week 52 (W52) and week 92 (W92), corresponding approximately to embryo, newborn, youth, adolescence, adult and old age in humans(7). Partitioning this data by age, we generated entropy metrics and then clustered genes by their $\psi_{\text{block}}$ scores (Fig. 2g). This analysis revealed a cluster of genes specific to old age with high specificity to W92 and slightly lower specificity to W52 (Cluster 10, 486 genes, Supp. table 9). A gene set enrichment analysis using the molecular signatures database (MSigDB) on this cluster of genes revealed heightened immune activity (Supp. table 10) (58). This is in agreement with findings from the Chen *et al*. 2025 authors, who found steadily increasing immune cell proportions from W12 to W92, indicating an accumulation of immune cells in kidneys upon aging (7). One gene in this cluster of *“aging-specific genes”* is *Igkc*, which encodes immunoglobulin kappa constant. *Igkc* has previously been identified as an aging marker across several tissues, including kidney, in a proteomics study comparing 32 and 72 week old mice (59).

We found a cluster of genes specific to non-consecutive developmental stages. Cluster 22, a cluster of 222 genes, shows specificity to ages P0, W52 and W92. A gene set enrichment analysis of this cluster using MsigDB reveled heightened immune and inflammatory response (Supp. table 9, 11) (58). A study by Speer *et al*. 2025 found that neonatal sepsis lead to increased expression of renal tissue inflammation and injury biomarkers, consistent with acute kidney injury, and drawing some parallels between the molecular signatures of a new born mouse to that of an aged one (60), hinting at that biology that may be underlying this unique non-consecutive bimodal expression. Secretory leukocyte protease inhibitor (*Slpi*), is a gene within this cluster of genes specific to new borns and old age, plays a regenerative role in the ischemic renal (I/R) injury model, through activation induced by *MIF-2/D-DT* (61). Together, these results show that applying unsupervised learning techniques to entropy metrics provides an unbiased methodology for identify clusters of genes with distinct and unexpected expression patterns.

### Tissue and cell type are the dominant source of biological variation in a scRNAseq experiment.

#### Tissue-linked specificity.

**Fig. 3.**
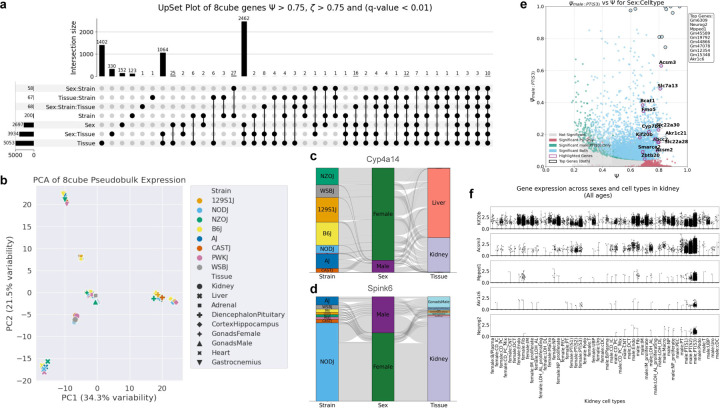
Visualizing specificity patterns across partitions **a.** Upset plot generated from 8cube dataset (6) selecting highly specific genes partitioned by sex, strain, tissue and their 2-way and 3-way interaction terms. Thresholds used for highly specific gene selection are Ψ>0.75 and ζ>0.75. Global testing correction was performed across all 7 partitioned and genes selected passed a significance threshold of 0.01. **b.** PCA plot of 8cube data pseudo-bulked by strain (color) and tissue (shape) (6). **c, d.** Alluvial plots depicting pseudo-bulked gene expression of *Cyp4a14* and *Spink6* across strains, sexes and tissues in 8cube (6), generated using wompwomp (25). **e.** Scatter plot showing gene specificity to male proximal tubule epithelial cells (Segment 3) in developmental kidney (7), partitioned by sex:celltype. Genes with high Ψ and high $\psi_{\text{male}PT(S3)}$ are identified as marker genes (highlighted in black). Top differentially expressed genes in male PT(S3) cells identified by Chen *et al*. 2025 (7) are highlighted in purple. Significance testing via permutation testing for both metrics is shown by color: genes significant for both are light blue and exhibit reliable specificity. Top marker genes are listed in the top-right text box. **f.** Violin plots for *Kif20b* and *Acsm3*, identified as differentially expressed in male PT(S3) cells by (7) and *Mpped1*, *Akr1c6* and *Neurog2*, identified by ember as highly specific to male PT(S3) cells.

Generating entropy metrics for multiple partitions in a dataset allows unique insights into biological patterns and trends. We generated Ψ and ζ values partitioning the entire 8cube data by tissue, sex and mouse strain and their two and three way interaction terms (6). We extracted lists of genes most specific to these partitions (Ψ>0.75,ζ>0.75) and from these seven sets of genes, we generated an upset plot to visualize gene membership (Fig. 3a) (62). We found genes to be most specific to tissue, along with a large set of genes with sex specificity linked to tissue. We also found that tissue specificity dominates strain specificity, with only 124 genes highly specific to strain and 1401 genes highly specific to tissue. To further validate this observed pattern, we performed a principal component analysis of gene expression data pseudo-bulked by mouse strain and tissue. PC1 and PC2 together account for more than half of the total variability (55.8% of total variance, 34.3% and 21.5% for PC 1 and 2 respectively), indicating that the major biological factors driving variation are captured effectively within the first two principal components (Fig. 3b). The pseudo-bulked expression profiles show distinct clustering by tissue (shape), over mouse strain (color). In addition, a correlation cluster map of this pseudo-bulked data also revealed that tissues across strains had higher correlation to one another (Supp. Fig 3). These results show that tissue is the dominant source of biological variation, making genes with specificity to partitions other than tissue especially interesting.

The upset plot in Fig 3a isolates *Cyp4a14*, which encodes a cytochrome P450 monooxygenase, to be the only gene highly specific to sex:strain:tissue over other partitions (Fig. 3c). *Cyp4a14* shows biased expression towards female liver and kidneys in all strains except PWKJ, which as we have previously noted, are the most prone of the founder strains to develop hepatic inflammation and progress to fibrosis of the founder strains (57). Incidentally, it has been documented that *Cyp4a14* is implicated in the progression of hepatic fibrosis and that over-expression in a bile duct ligation murine model exacerbates fibrosis, while a knockout attenuates it (63). In this analysis we also found *Spink6* to be the only gene highly specific to tissue:strain over other partitions (Fig 3d). *Spink6* encodes serine peptidase inhibitor kazal type 6 and shows biased expression towards NODJ kidney.

#### Cell-type-linked specificity.

We observe a similar pattern at the tissue level, where a large proportion of biological variation within a tissue is explained by cell type rather than strain, 38.8% in 8cube kidney (6). In the developing kidney (7), we see that specificity by cell type dominates specificity by age and sex. (Supp. Fig 3a, b, c). In a tissue like the kidney, where it is interesting to study sexual dimorphism, being able to resolve specificity to sex from specificity to sex within a cell type is critical (64). Chen *et al*. 2025 identified that proximal tubule epithelial cells, specifically segment 3 (PTS3) present the largest number of sexually dimorphic genes (7). Chen *et al*. 2025 identified genes as sexually dimorphic in PTS3 cells with biased expression towards males using a non-parametric Wilcoxon rank sum through the Surat package.

We re-analyzed the developmental kidney dataset using ember to understand male-biased sexual dimorphism in PTS3 cells (7). We generated entropy metrics, partitioning the data by sex:celltype and overlayed the top genes identified by Chen *et al*. 2025 on a scatter plot showcasing specificity to male PTS3 cells (Ψ vs. $\psi_{\text{male:}PTS3}$). We noticed that these genes had Ψ>0.6, and had a spread of $\psi_{\text{male:}PTS3}$ scores lower than 0.65 (Fig. 3e), indicating that while these genes may have high expression in male PTS3s, they are not truly specific to the block. Upon closer inspection we found that one of the genes on this list, *Kif20b* along with having significant expression across various cell types, has biased expression towards males in PTS3 cells but towards females in loop of henle ascending limb proliferating cells, making it specific to neither sex nor cell type (Fig. 3f). We found that another gene on this list *Acsm3*, has high expression not only in male PTS3 cells but also in male PTS1 and male PTS2 (Fig 3f). Conversely, genes with high Ψ and $\psi_{\text{male:}PTS3}$ scores identified by ember show distinct and exclusive specificity to male PTS3 cells (Fig 3f). These results re-emphasize the advantage of leveraging decomposable gene-specificity when finding differentially expressed genes in a multivariate dataset.

#### Variation in humans.

**Fig. 4.**
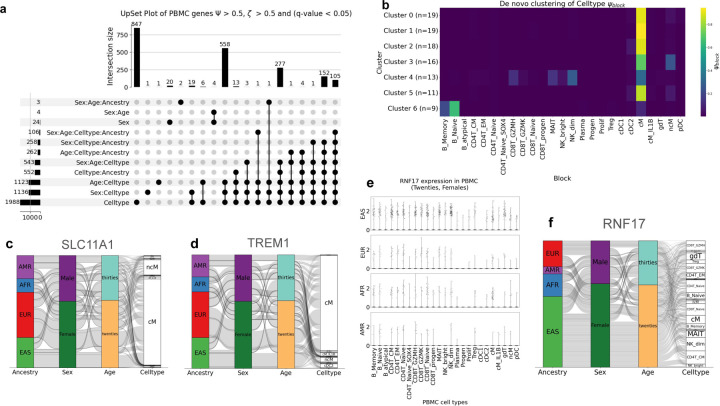
Specificity in human PBMCs collected from 255 individuals from diverse ancestriesa. Upset plot generated from human PBMC dataset selecting highly specific genes partitioned by Sex, Age, Ancestry and Celltype and their 2-way, 3-way and 4-way interaction terms. Thresholds used for highly specific gene selection are Ψ>0.5 and ζ>0.5. Global testing correction was performed across all 7 partitions and genes selected passed a significance threshold of 0.05. **c, d, f.** Alluvial plots depicting pseudo-bulked gene expression of *SLC11A1*, *TREM1* and *RNF17* respectively across ancestries, sexes, ages and cell types in human PBMCs. **e.** Violin plots of *RNF17* gene expression in 20–29 year-old females across ancestries and cell types in human PBMCs.

We generated entropy metrics for a human PBMC dataset of 255 individuals from four different ancestries: European, African, East Asian and Admixed American, partitioning by age, cell type, sex, ancestry and their two-way, three-way and four-way interaction terms (Fig 4a). We found, similar to mouse, that variation within PBMCs was dominated by cell type and that sex, age and ancestry related specificity are mostly linked to cell type. 105 genes were identified as specific to cell type or a cell-type-linked interaction term (Supp. table 12). We clustered these 105 genes on their $\psi_{\text{block}}$ scores and found that not only is cell type the dominant source of variation in PBMCs, but also this variation can be largely localized to classical monocytes (Fig. 4b). One of the 105 highly variable genes is *SLC11A1*, which encodes a member of the solute carrier family 11, a family of proton-coupled divalent metal ion transporters (Fig. 4c). Mutations in *SLC11A1* have been associated with susceptibility to diseases such as tuberculosis in Chinese population (65), kawasaki disease in Japanese population (66), cutaneous leishmaniasis in Pakistani population (67), type 2 diabetes mellitus in Iranian population (68) and many other ancestry specific variants that offer resistance or heightened susceptibility to disease (69).

Another gene in this list of 105 highly variable genes is *TREM1*, which encodes triggering receptor expressed on myeloid cells 1 and is an ancestral predictor for malaria susceptibility (Fig. 4d). A study of Columbian population showed lower malaria susceptibility in people of Native American ancestry, slightly higher susceptibility in European ancestry and oscillated between a protective effect and increased risk with no clear prediction in African ancestry (70). Another gene *ILB1* is on their list of ancestral malaria predictor genes as well as on our list of 105 highly variable genes linked to cell type.

Outside of cell-type-linked specificity, we found 20 genes uniquely specific to sex and 4 genes specific to sex:age, of which 18 were Y-chromosome-linked genes and the remaining 6 were X-chromosome-linked. We additionally found one gene, *RNF17*, which showed distinct specificity by sex:age:ancestry (Fig. 4e, f). *RNF17* encodes a protein containing a RING finger domain and has been previously considered testes specific. We found that *RNF17* shows biased expression in East Asian ancestry females in their twenties in human PBMCs.

Together, these results show that an entropy-guided approach is effective in understanding biological variance in human genetic data and can find known and novel partition-specific and highly variable genes. This analysis also draws to attention some limitations of the mouse model in understanding genetic variation in humans (24). We identified strain-specific genes across all tissues as well as within a tissue in the mouse 8cube data (6) (Fig. 1a), but such a parallel does not exist in humans. There are no genes that are exclusively ancestry-specific in human PBMCs. Instead, in humans we observe that ancestry specificity is linked to sex and cell type.

## Discussion

Lists of marker genes or differentially expressed genes are often important takeaways from a scRNAseq experiment, but current methods lack a homogeneous definition of what constitutes a marker gene. An idealistic marker gene for a block in a partition should have distinct and exclusive expression in this block. Yet, even the long-noncoding RNA *Xist/XIST*, which is indispensable for X chromosome inactivation during early development in both humans and mice, has non-zero expression in normal male tissues (71). This brings to question whether such idealism, when trying to understand the complexities of biology, may prevent us from truly learning what the transcriptomic orchestra of scRNAseq has to say.

A similar argument can be made for the definition of a housekeeping gene. An ideal housekeeping gene has been described as stably expressed irrespective of tissue type, developmental stage, cell cycle state, or external signal (72, 73). However, the inherently stochastic and bursty nature of transcription (74, 75) undercuts the existence of such a perfectly invariant housekeeping gene. Taking a more nuanced and context-aware approach to marker gene and housekeeping discovery, that accounts for the limitations of an experiment, such as tissues sampled, mouse strain used, sexes sampled, human populations sampled, and rarity and resolution of a cell type, may lead to more reproducible biological insights. Our tool ember’s unbiased entropy-based approach, along with its attention to multivariate partitioning of a dataset allow for discovery of nuanced marker and housekeeping genes, quantifying specificity without assuming exclusivity.

A further challenge to interpreting nuanced gene expression lies in the availability of appropriate visualization tools. A violin plot allows for visualization at single-cell resolution, but is constrained to display expression within a partition. Alluvial plots allow for effective visualization of a gene’s expression across multiple partitions, though at the cost of single-cell resolution (25, 76). To balance these trade-offs, we use both alluvial and violin plots in tandem. This complementary approach allowed us to highlight genes with intricate specificity uncovered by ember, providing interpretable and biologically meaningful visualizations. More broadly, our work aligns with critiques of visualization in single-cell genomics (77), emphasizing that the interpretability of biological results is inseparable from the limitations and choices of visualization strategy.

Our work provides a methodology to interpret gene specificity that is distribution agnostic, decomposable, robust to replicates and that fully leverages single-cell resolution, but it is not without its limitations. First, ember requires discrete partitioning of cells into blocks and is not equipped to deal with continuous variables. We recommend binning of continuous variables into biologically meaningful categories to get an approximate estimate of related gene specificity. Second, while ember is sensitive to lowly expressed genes, transcripts detected in very few cells yield unstable entropy estimates. As a result, filtering is required to remove genes expressed in fewer than 100 cells, which improves robustness but may inadvertently exclude biologically relevant or rare transcripts. Finally, computation of entropy metrics and p-values through sampling can be time consuming when dealing with dense data that have a large number of cells (>1000000 cells). We recommend using parallel computation and chunking strategies where possible to make analyses more manageable. Collectively, these constraints highlight areas for future methodological improvement.

When quantifying gene specificity in scRNA-seq experiments, a central challenge lies in placing results within a broader biological context. Frameworks that enable generalizable conclusions are essential for ensuring accessibility and reproducibility. Our entropy-based approach is particularly well-suited to revealing such organism-level patterns, distilling high-dimensional single-cell data into interpretable, large-scale conclusions. From our analyses, we found that the dominant source of biological variation within an organism is tissue or cell type. Specifically in the 8cube dataset, we conclude that tissue specificity outweighs genotype specificity. These findings align with conclusions made by Gilad and Mizrahi-Man in their reanalysis of the ENCODE data, that variability of genes by tissue is greater than variability by species (78). Taken together, these results reinforce the view that tissue is a primary driver of transcriptomic specificity.

Despite this broader pattern of specificity, it is wise to exercise caution when drawing cross-strain and cross-species conclusions. We observe in our analysis of the 8cube data (6) that there are many distinct differences in gene expression between strains. Strain-specific diversity limits the extent to which findings can be generalized. The heavy reliance on inbred strains exacerbates this issue, raising the possibility that observed specificity reflects artifacts of restricted genetic backgrounds rather than broadly conserved mechanisms. These concerns emphasize the danger of extrapolating from a single strain to complex, heterogeneous populations. We have deposited specificity and gene expression information across the 8 diverse founder strains into a publicly available and programmaticaly accessible API database and website (Data Availability), fueling the effective and informed use of mouse models in biological research.

An important evolutionary question emerges from these observations: how do highly specific genes arise and change across lineages? Is there an adaptive advantage to maintaining a housekeeping role, or can specificity confer selective benefits? Moreover, how can a gene that is highly specialized in one mouse strain acquire a different function and specificity in another? The evolutionary trajectories of marker and housekeeping genes remain poorly understood (12, 79, 80). Addressing these questions may provide key insights into the origins of specificity and the selective pressures shaping gene expression programs across species. By providing a robust and quantitative framework for measuring and dissecting gene specificity, ember offers a way to begin tracing such evolutionary trajectories, enabling comparative analyses of how marker and housekeeping functions emerge, shift, and are maintained across contexts.

## Methods

### Ψ, ζ, and $\psi_{\text{block}}$ scores.

Let *x*_*i*_ be the count of a gene in cell i. Define the probability

$$p_{i}=\frac{x_{i}}{\sum_{n}x_{j}},$$

i.e., the proportion of the gene’s total count found in cell i.

The **total entropy** $E_{T}$ of the gene expression across all cells is defined as:

$$E_{T}=-\sum_{i=1}^{n}p_{i}\text{log}p_{i},$$

where n is the number of cells.

Let n cells be partitioned into r blocks. We define Ψ based on the within-block and between-block decomposition of entropy. And let $q_{jC}$ be the proportion of expression in cell j relative to the total in block C:

$$q_{jC}=\frac{x_{j}}{\sum_{k\in C}x_{k}}\;\text{for}\;j\in C$$

Then, $E_{C}$ is the **entropy within the block C**:

$$E_{C}=-\sum_{j\in C}q_{jC}\;\text{log}\;q_{jC}.$$

The total **within entropy** $E_{W}$ is the weighted sum:

$$E_{W}=\sum_{C}p_{C}E_{C}.$$

Since the total entropy $E_{T}$ can be decomposed as:

$$E_{T}=E_{B}+E_{W},$$

Then, we define Ψ **(Psi) as the fraction of information explained by a partition** of n cells:

$$\Psi =1-\frac{E_{B}}{E_{T}}=\frac{E_{W}}{E_{T}}.$$

We define the decomposable elements of $E_{W}$ for each block as $\psi_{\text{block}}$, a measure of **specificity to a block**. Then $\psi_{\text{block}}$ for block C is given by:

$$\psi_{\text{block}}=\frac{E_{C}}{E_{W}}.$$

Let ($\psi_{\text{blocks}}$) denote the set of $\psi_{\text{block}}$ scores across all (r) blocks:

$$\psi_{\text{blocks}}=\psi_{\text{block}}^{\left(C\right)}:C=1,\ldots ,r.$$

We define ζ as the normalized Kullback-Leibler divergence of the distribution of $\psi_{\text{blocks}}$ to the uniform distribution. This metric quantifies **specificity to a partition**.


$$\zeta =1-\frac{H\left(\psi_{\text{blocks}}\right)}{\text{log}\;r}$$

### Sampling over replicates.

To ensure balanced representation of replicates across categories in an experimental design, we implemented a sampling strategy that generates subsets of samples with equal representation of experimental conditions within each category. For every unique category (e.g., genotype, strain, or age group), replicates were iteratively selected so that each condition (e.g., sex) was represented exactly once per sampling draw. The distinction between “category” and “condition” was made for conceptual clarity, although these variables can be defined interchangeably. Sampling was performed at the level of unique sample identifiers recorded in the sample metadata (e.g., mouse_ids or sample_ids). Multiple balanced subsets (typically 100 draws) were generated while minimizing repeated selection of the same samples across draws by tracking sample usage frequency. Random seeds were used to ensure reproducibility, and the approach enforced complete balance by requiring at least one sample for every category-condition pair. (Supp. Fig 5 a,b,c)

Arithmetic mean and standard deviation values for Ψ and ζ were calculated to capture variation in specificity across biological replicates.

Since $\psi_{\text{block}}$ values represent compositional fractions that sum to one within each partition, we aggregated replicate level $\psi_{\text{block}}$ estimates across sampling draws using compositional data analysis principles. Specifically, the Aitchison mean was used to compute the central tendency of $\psi_{\text{block}}$ values across draws, while the geometric standard deviation quantified variability in block-specific specificity estimates.

### Octagon plot representation of block specificity.

The octagon plot from Fig 1a was designed to visualize variation in specificity across biological replicates to the 8 different founder mouse strains. Because $\psi_{\text{block}}$ values for a given partition represent compositional fractions that sum to one, gene-specificity profiles can be represented as points within a simplex. In this framework, each coordinate corresponds to the proportion of a gene’s specificity attributed to a particular block, such that all coordinates are non-negative and collectively sum to unity. In general, an n-simplex can be projected onto a regular (n+1)-gon, yielding n!/2 unique 2D projections. For visualization of strain-level specificity across the eight founder mouse strains, the geometry of the 7-simplex was projected into two dimensions as one of 2,540 possible regular octagonal representations. We chose the projection ordered by distance in single nucleotide variants to B6J mice. Data used to generate this plot is available at the supplementary data link (Supplementary Information). Code to generate this compositional octagon plot is in the Code Availability section.

### Generating p-values and q-values.

Significance of $\Psi ,\psi_{\text{block}}$, and ζ values were estimated by comparing observed mean values after sampling to null distributions generated from scrambling block labels. For each gene, empirical p-values were computed as the proportion of permuted values greater than or equal to the observed statistic.

All resulting p-values were combined across metrics and subjected to global false discovery rate (FDR) correction using the Benjamini–Hochberg procedure to obtain q-values.

### Interpreting specificity from entropy metrics.

To identify highly and lowly specific genes, we applied a standardized workflow consisting of data pre-processing, entropy metric calculation, and statistical filtering.

#### Pre-processing steps.

For all three datasets analyzed, genes expressed in fewer than 100 cells were removed. Count data was normalized by the original data generators using the default normalization workflows implemented in Scanpy and Seurat (81, 82).

#### Entropy metric workflow.

Entropy-based specificity metrics were calculated using the ember Python package (see Code Availability for repository link). Gene-level entropy metrics were first computed for each dataset, after which genes with q-values<0.05 were retained. Genes were then ranked according to their respective entropy metrics, and biologically meaningful thresholds (default 0.5 if no prior knowledge) were applied to identify the most specific genes within each partition or block.

##### (a) Specific to a block / “Marker” genes

Metrics $\psi_{\text{block}}$ and Ψ were used to identify genes with high specificity to individual blocks. Genes with the highest $\psi_{\text{block}}$ and Ψ values were considered most specific to the selected block (e.g., skeletal muscle satellite cells in gastrocnemius, Fig. 1b).

##### (b) Specific to a partition

Metrics ζ and Ψ were used to identify genes specific to a partition. Genes with the highest ζ and Ψ values were interpreted as most specific to the partition (e.g., gastrocnemius partitioned by cell type, Fig. 1c).

##### (c) Non-specific to a partition / “Housekeeping” genes

Metrics ζ and Ψ were also used to identify non-specific or “housekeeping” genes. Genes with low ζ but high Ψ values were interpreted as least specific to any single partition (e.g., gastrocnemius partitioned by cell type, Fig. 1c).

### Finding specificity trends using upset plots.

To identify large-scale trends in gene specificity across multiple partitions, we computed Ψ and ζ scores for all relevant partitions, including interaction terms (e.g., two-way, three-way, or four-way combinations). Genes with q-value<0.05 for both metrics were selected. Genes exhibiting strong specificity within each partition were selected using a biologically informed or default cutoff of 0.5 when no prior information was available. The sets of highly specific genes from each partition were then combined to define gene memberships, which were visualized using upset plots to reveal overlaps and sets of genes with shared specificity patterns across partitions (62). Data used to generate upset plots is available at the supplementary data link (Supplementary Information). Code to generate upset plots is in the Code Availability section.

## Supplementary Material

Supplement 1

Supplement 2

Supplement 3

## Figures and Tables

**Table 1.** Clusters of genes from Fig 2a. with specificity to male gonads and one other tissue.

Tissue	Cluster	Gene Count	Functional EnrichmentSummary
Female Gonads	31	136	mitosis, chromosome segregation, reproductive processes
Kidney	24	226	Regulated by twist motif, peptidase activity, serine-type peptidase activity, serine hydrolase activity
Cortex, Hippocampus	27	208	Cell signaling, membrane receptor activity, GPCR activity, olfactory receptor activity
Diencephalon, Pituitary	18	363	Sensory perception, response to chemical stimulus, GPCR activity, olfactory receptor activity
Gastrocnemius	36	53	None

## Data Availability

Mouse specificity explorer website for visualizing specificity and gene expression in the 8cube data (6): https://mouseexplorer.onrender.com. 8cubeDB API documentation: https://eightcubedb.onrender.com/docs. 8cubeDB API and mouse explorer website source code: https://github.com/pachterlab/8cubeDB. ember python package and documentation: https://github.com/pachterlab/ember.

## Code availability.

The code for reproducing the figures and analyses: https://github.com/pachterlab/SBRGKLAYWMP_2025. Data to reproduce the figures and analyses: https://doi.org/10.5281/zenodo.17654220.
