# Supplementary material for: Determining gene specificity from multivariate single-cell RNA sequencing data
Source: bioRxiv. 2025 Nov 23:2025.11.21.689845. Preprint. [Version 1] doi: 10.1101/2025.11.21.689845 (PMC12667802; doi:10.1101/2025.11.21.689845)
Supplement: Supplement 2 [file media-2.pdf]

Supplementary figures

Determining gene specificity from multivariate single-cell RNA sequencing data

Nikhila P. Swarna[1], A. Sina Booeshaghi[3], Elisabeth Rebboah[4,5], M. Grace Gordon[6,7,8,9], Pooja Kathail[10], Taibo Li[11], Marcus Alvarez[8], Chun Jimmie Ye[7,8,9,12,13,14,15,16], Barbara Wold[1], Ali Mortazavi[4,5], and Lior Pachter[1,2,*]

[1]Division of Biology and Biological Engineering, California Institute of Technology, Pasadena, CA, USA
[2]Department of Computing and Mathematical Sciences, California Institute of Technology, Pasadena, CA, USA
[3]Department of Bioengineering, University of California at Berkeley, Berkeley, CA, USA
[4]Department of Developmental and Cell Biology, University of California at Irvine, Irvine, CA, USA
[5]Center for Complex Biological Systems, University of California Irvine, Irvine, CA, USA
[6]Biological and Medical Informatics Graduate Program, University of California, San Francisco, CA, USA
[7]Division of Rheumatology, Department of Medicine, University of California, San Francisco, CA, USA
[8]Institute for Human Genetics, University of California, San Francisco, CA, USA
[9]Department of Bioengineering and Therapeutic Sciences, University of California, San Francisco, CA, USA
[10]Center for Computational Biology, University of California, Berkeley, Berkeley, CA, USA
[11]Department of Biomedical Engineering, Johns Hopkins University, Baltimore, MD, USA
[12]Gladstone-UCSF Institute of Genomic Immunology, San Francisco, CA, USA
[13]Department of Epidemiology and Biostatistics, University of California, San Francisco, San Francisco, CA, USA
[14]Bakar Computational Health Sciences Institute, University of California,

San Francisco, San Francisco, CA, USA
[15]Parker Institute for Cancer Immunotherapy, University of California, San Francisco, San Francisco, CA, USA
[16]Arc Institute, Palo Alto, CA, USA
*To whom correspondence should be addressed: lpachter@caltech.edu

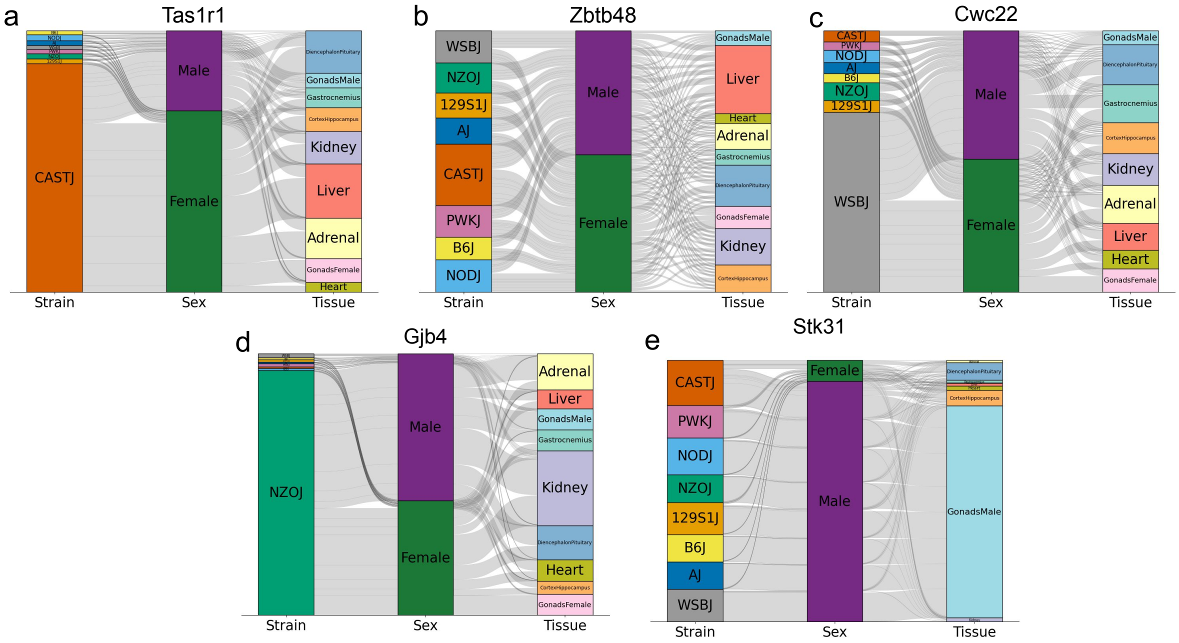

Figure 1: **Alluvial plots showing pseudo-bulked expression across partitions of strain-specific genes a.** *Tas1r1* a CASTJ specific genes. **b.** *Zbtb48*, a gene upstream of *Tas1r1* with slight bias towards CASTJ. **c.** *Cwc22*, a WSBJ specific gene [2]. **d.** *Gjb4*, a NZOJ specific gene [3]. **e.** *Stk31*, a gene that displays strain driven cell type switching in gastrocnemius tissue.

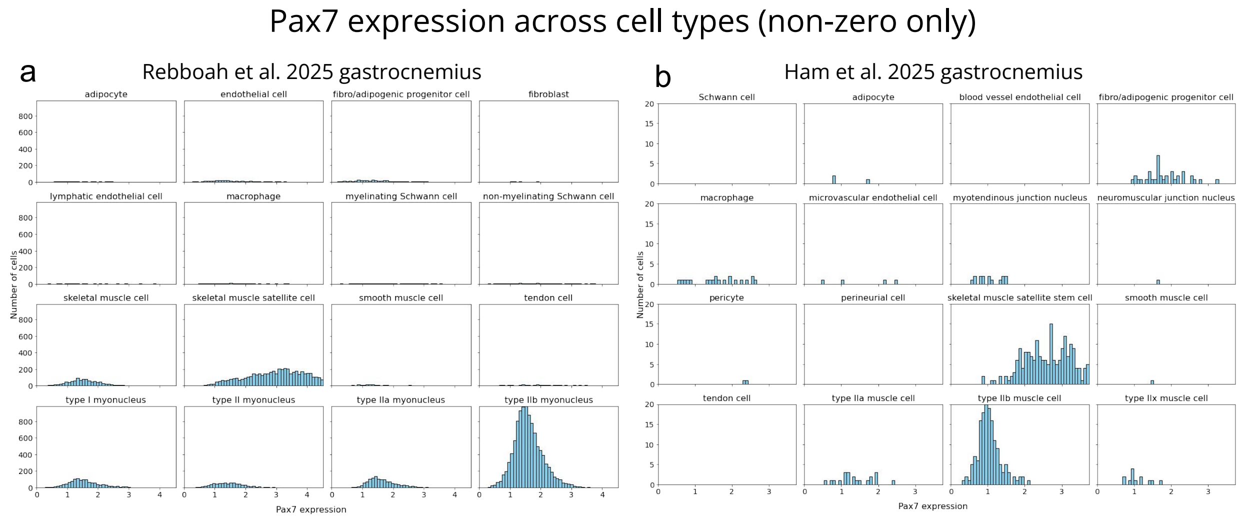

Figure 2: **Pax7 expression across cell types** Histograms of sequence depth and log1p normalized counts across gastrocnemius cells for *Pax7* in **a.** 8cube Rebboah et al. 2025 [5] and **b.** Ham et al. 2025 [4]

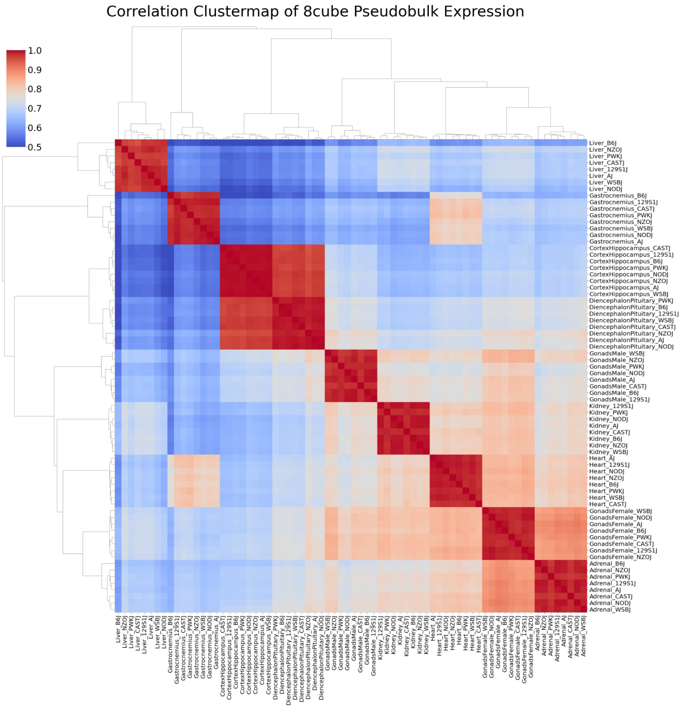

Figure 3: **Correlation cluster map of 8cube data pseudo-bulked by strain and tissue** [5]

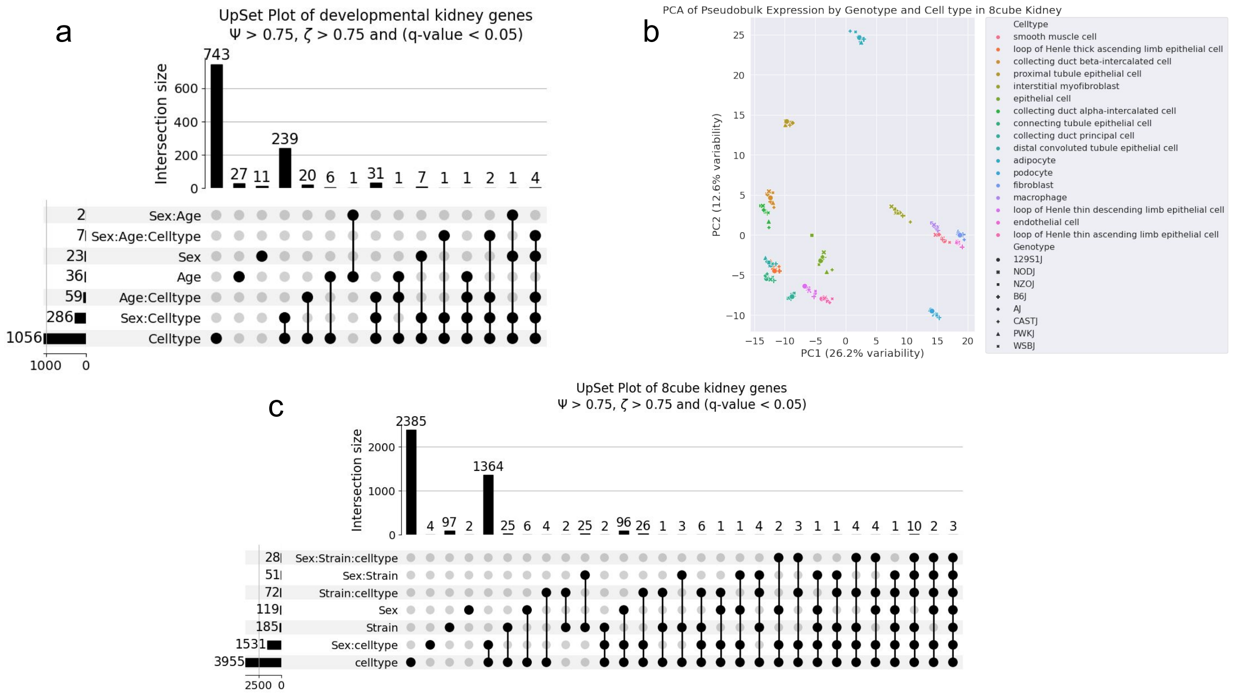

Figure 4: **Specificity trends in the kidney a.** Upset plot of developmental kidney specificity generated from Chen et al. 2025 dataset [1]. We selected highly specific genes partitioned by Sex, Age, Celltype and their 2-way and 3-way interaction terms. Thresholds used for highly specific gene selection are $\Psi > 0.75$ and $\zeta > 0.75$. Global testing correction was performed across all 7 partitioned and genes selected passed a significance threshold of 0.05. **b.** PCA plot of 8cube kidney data pseudo-bulked by cell type (color) and strain(shape) [5]. **c.** Upset plot of 8cube kidney specificity [5]. We selected highly specific genes partitioned by Sex, Strain, Celltype and their 2-way and 3-way interaction terms. Thresholds used for highly specific gene selection are $\Psi > 0.75$ and $\zeta > 0.75$. Global testing correction was performed across all 7 partitioned and genes selected passed a significance threshold of 0.05.

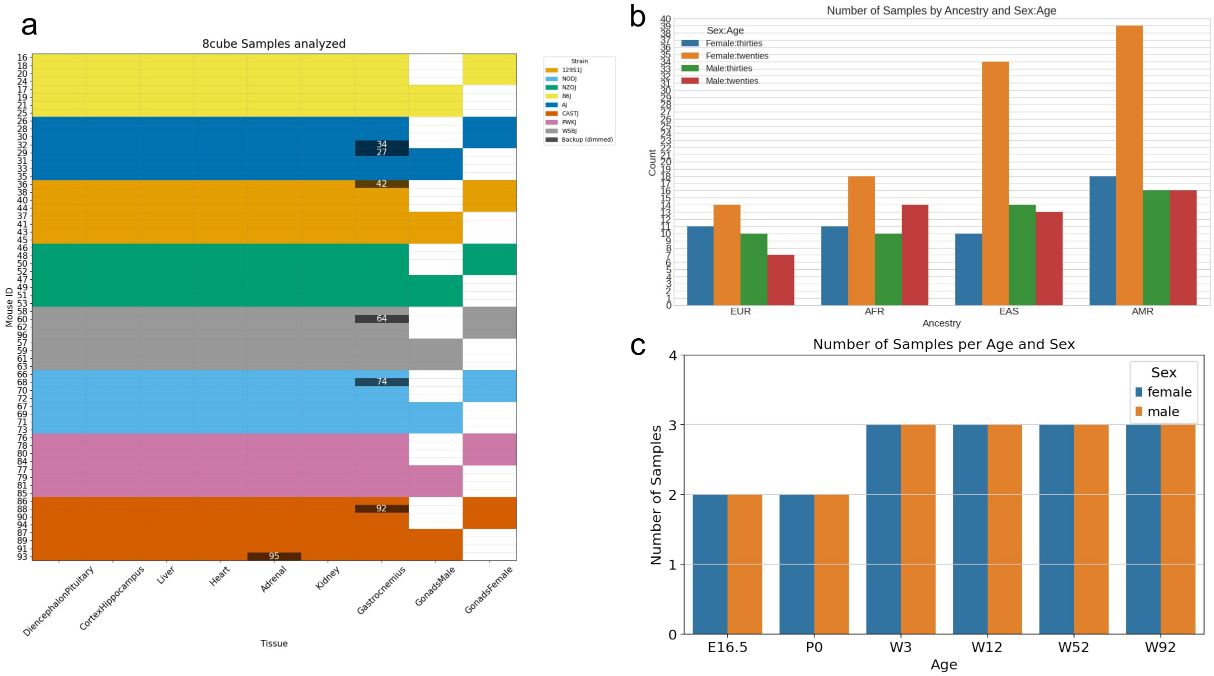

Figure 5: **Biological replicates analyzed from each dataset a.** Samples analyzed from 8cube data, colored by strain. Replacement tissue samples from additional mice shaded in black. **b.** Number of samples analyzed from human PBMCs collected from 255 diverse individuals, grouped by sex, ancestry and age (binned as twenties and thirties). **c.** Number of biological replicates analyzed from developmental kidney data [1], grouped by sex and age.

References

[1] Siqi Chen, Ruiyang Liu, Chia-Kuei Mo, Michael C. Wendl, Andrew Houston, Preet Lal, Yanyan Zhao, Wagma Caravan, Andrew T. Shinkle, Atieh Abedin-Do, Nataly Naser Al Deen, Kazuhito Sato, Xiang Li, André Luiz N. Targino da Costa, Yize Li, Alla Karpova, John M. Herndon, Maxim N. Artyomov, Joshua B. Rubin, Sanjay Jain, Xue Li, Sheila A. Stewart, Li Ding, and Feng Chen. Multi-omic and spatial analysis of mouse kidneys highlights sex-specific differences in gene regulation across the lifespan. *Nature Genetics*, 57:1213–1227, 04 2025.

[2] John P. Didion, Andrew P. Morgan, Amelia M.-F. Clayshulte, Rachel C. McMullan, Liran Yadgary, Petko M. Petkov, Timothy A. Bell, Daniel M. Gatti, James J. Crowley, Kunjie Hua, David L. Aylor, Ling Bai, Mark Calaway, Elissa J. Chesler, John E. French, Thomas R. Geiger, Terry J. Gooch, Theodore Jr. Garland, Alison H. Harrill, Kent Hunter, Leonard McMillan, Matt Holt, Darla R. Miller, Deborah A. O'Brien, Kenneth Paigen, Wenqi Pan, Lucy B. Rowe, Ginger D. Shaw, Petr Simecek, Patrick F. Sullivan, Karen L. Svenson, George M. Weinstock, David W. Threadgill, Daniel Pomp, Gary A. Churchill, and Fernando Pardo-Manuel de Villena. A multi-megabase copy number gain causes maternal transmission ratio distortion on mouse chromosome 2. *PLoS Genetics*, 11(2):e1004850, 02 2015.

[3] Anneke Gässler, Charline Quiclet, Oliver Kluth, Pascal Gottmann, Kristin Schwerbel, Anett Helms, Mandy Stadion, Ilka Wilhelmi, Wenke Jonas, Meriem Ouni, Frank Mayer, Joachim Spranger, Annette Schürmann, and Heike Vogel. Overexpression of gjb4 impairs cell proliferation and insulin secretion in primary islet cells. *Molecular Metabolism*, 41:101042, 06 2020.

[4] Alexander S. Ham, Shuo Lin, Alice Tse, Marco Thürkauf, Timothy J. McGowan, Lena Jörin, Filippo Oliveri, and Markus A. Rüegg. Single-nuclei sequencing of skeletal muscle reveals subsynaptic-specific transcripts involved in neuromuscular junction maintenance. *Nature Communications*, 16:2220, 03 2025.

[5] Elisabeth Rebboah, Ryan Weber, Elnaz Abdollahzadeh, Nikhila Swarna, Delaney K. Sullivan, Diane Trout, Fairlie Reese, Heidi Yahan Liang, Ghassan Filimban, Parvin Mahdipoor, Margaret Duffield, Romina Mojaverzargar, Erisa Taghizadeh, Negar Fattahi, Negar Mojgani, Haoran Zhang, Rebekah K. Loving, Maria Carilli, A. Sina Booeshaghi, Shimako Kawauchi, Ingileif B. Hallgrímsdóttir, Brian A. Williams, Grant R. MacGregor, Lior Pachter, Barbara J. Wold, and Ali Mortazavi. Systematic cell-type resolved transcriptomes of 8 tissues in 8 lab and wild-derived mouse strains captures global and local expression variation. *bioRxiv*, 04 2025. Preprint, not peer reviewed.
