# Supplementary material for: Determining gene specificity from multivariate single-cell RNA sequencing data
Source: bioRxiv. 2025 Nov 23:2025.11.21.689845. Preprint. [Version 1] doi: 10.1101/2025.11.21.689845 (PMC12667802; doi:10.1101/2025.11.21.689845)
Supplement: Supplement 3 [file media-3.pdf]

Supplementary Note - Psi Uniqueness Theorem

Determining gene specificity from multivariate single-cell RNA sequencing data

Nikhila P. Swarna[1], A. Sina Booeshaghi[3], Elisabeth Rebboah[4,5], M. Grace Gordon[6,7,8,9], Pooja Kathail[10], Taibo Li[11], Marcus Alvarez[8], Chun Jimmie Ye[7,8,9,12,13,14,15,16], Barbara Wold[1], Ali Mortazavi[4,5], and Lior Pachter[1,2,*]

[1]Division of Biology and Biological Engineering, California Institute of Technology, Pasadena, CA, USA

[2]Department of Computing and Mathematical Sciences, California Institute of Technology, Pasadena, CA, USA

[3]Department of Bioengineering, University of California at Berkeley, Berkeley, CA, USA

[4]Department of Developmental and Cell Biology, University of California at Irvine, Irvine, CA, USA

[5]Center for Complex Biological Systems, University of California Irvine, Irvine, CA, USA

[6]Biological and Medical Informatics Graduate Program, University of California, San Francisco, CA, USA

[7]Division of Rheumatology, Department of Medicine, University of California, San Francisco, CA, USA

[8]Institute for Human Genetics, University of California, San Francisco, CA, USA

[9]Department of Bioengineering and Therapeutic Sciences, University of California, San Francisco, CA, USA

[10]Center for Computational Biology, University of California, Berkeley, Berkeley, CA, USA

[11]Department of Biomedical Engineering, Johns Hopkins University, Baltimore, MD, USA

[12]Gladstone-UCSF Institute of Genomic Immunology, San Francisco, CA, USA

[13]Department of Epidemiology and Biostatistics, University of California, San Francisco, San Francisco, CA, USA

[14]Bakar Computational Health Sciences Institute, University of California, San Francisco, San Francisco, CA, USA

[15]Parker Institute for Cancer Immunotherapy, University of California, San Francisco, San Francisco, CA, USA

[16]Arc Institute, Palo Alto, CA, USA

[*]To whom correspondence should be addressed: lpachter@caltech.edu

1 Properties for a specificity measure

We posit that a specificity measure for single-cell genomics data should quantify how concentrated the expression of a gene is relative to a given partition of the data. Let A be a partition of the set of cells into groups (e.g. tissues, clusters, or sexes), and let

$$p = (p_1, \dots, p_n) \in \Delta_{n-1}, \quad \Delta_{n-1} = \{(p_1, \dots, p_n) \mid p_i \geq 0, \sum_i p_i = 1\},$$

denote the normalized expression profile of a gene across all cells. The coarse distribution over groups is denoted by $p_A = (p_a)_{a \in A}$, where $p_a = \sum_{i \in a} p_i$ is the total fraction of expression in group a. The function $\Psi(p; A)$ measures how specific the gene's expression is to particular groups in A: it should approach 1 if expression is concentrated in a single group and 0 if expression is uniform across groups. Ideally, a specificity measure Ψ should satisfy several natural properties. It should be insensitive to the ordering of categories, vary smoothly with the data, and behave coherently under hierarchical refinements such as tissues subdivided into cell types. We formalize these properties as follows:

A1 **Boundary conditions:** $\Psi(p; A) = 0$ when p is uniform within and across the groups in A, and $\Psi(p; A) = 1$ when all mass lies in a single group. More generally, if all mass is contained in one subgroup of a refinement $B \succ A$, then $\Psi(p; A, B) = 1$.

A2 **Symmetry:** $\Psi(p; A)$ is invariant under any permutation of indices within each group of A; that is, relabeling categories does not affect specificity.

A3 **Continuity:** Ψ is continuous on the probability simplex Δ_{n-1}: infinitesimal changes in p produce infinitesimal changes in $\Psi(p; A)$.

A4 **Additivity (Hierarchical decomposition):** For any refinement of partitions $A \prec B$, specificity satisfies the chain rule

$$\Psi(p; A) = \Psi(p; A, B) + \Psi(p; B \mid A),$$

where $\Psi(p; B \mid A)$ is a continuous, symmetric function of the conditional distributions $p(\cdot \mid a)$, satisfying the same boundary and symmetry properties within each group. No further structural assumptions on $\Psi(p; B \mid A)$ are imposed.

We show below that there is a unique function Ψ satisfying A1–A4. The proof is essentially Faddeev's proof [1] of the uniqueness of Shannon entropy (up to a constant). We recall Faddeev's theorem for completeness.

Theorem 1 (Faddeev, 1956 [1]). *Let $I : \Delta_{n-1} \to \mathbb{R}_{\geq 0}$ be a continuous, symmetric function satisfying the grouping axiom.*

$$I(p_{ij}) = I(p_i) + \sum_i p_i \, I(q_{j|i}), \quad \textit{where } p_{ij} = p_i q_{j|i},$$

and for each i, the conditional distribution $q_{j|i}$ satisfies

$$q_{j|i} = \frac{p_{ij}}{p_i}, \quad \sum_j q_{j|i} = 1.$$

Then there exists a constant $\kappa > 0$ *such that*

$$I(p) = \kappa\Big(-\sum_i p_i \log p_i\Big).$$

Theorem 2 (Uniqueness of Ψ via within/between decomposition)**.** *Under A1–A4,* Ψ *is uniquely determined and takes the form*

$$\Psi(p; A) = 1 - \frac{H(p_A)}{H(p)},$$

where $H(p) = -\sum_i p_i \log p_i$ *is the Shannon entropy.*

Proof. Define the functional

$$I(p) := 1 - \Psi(p; \text{discrete partition}),$$

where the discrete partition is $\{\{1\}, \dots, \{n\}\}$. By A1 and $0 \le \Psi \le 1$, we have $I(p) \ge 0$, and $I(p) = 0$ precisely when p is a point mass; by A2–A3, I is symmetric and continuous.

For any refinement of partitions $A \prec B$, A4 gives

$$\Psi(p; A) = \Psi(p; A, B) + \Psi(p; B \mid A).$$

To recover the recursion in Faddeev's theorem, take any partition $A = \{a_i\}$ with group masses $p_i = \sum_j p_{ij}$, and let B be its discrete refinement with probabilities $p_{ij} = p_i q_{j|i}$. Applying A4 to the refinement $A \prec B$ and using the definition $I(r) = 1 - \Psi(r; \text{discrete})$ at each level yields

$$I(p_{ij}) = I(p_i) + \sum_i p_i \, I(q_{j|i}),$$

which is exactly the grouping axiom in Faddeev's theorem. Therefore Faddeev's theorem implies

$$I(p) = c\, H(p) \quad \text{for some } c > 0.$$

Now consider an arbitrary partition A. Applying A4 to $A \prec B_{\text{disc}}$ (the discrete refinement of A) yields

$$I(p) = I(p_A) + \sum_{a \in A} p_a \, I\big(p(\cdot \mid a)\big).$$

Substituting $I = cH$ and using the Shannon chain rule

$$H(p) = H(p_A) + \sum_{a \in A} p_a \, H\big(p(\cdot \mid a)\big)$$

gives

$$cH(p) = cH(p_A) + \sum_{a \in A} p_a \, cH\big(p(\cdot \mid a)\big).$$

Rearranging and dividing by $cH(p) > 0$ yields the unique solution

$$\Psi(p; A) = 1 - \frac{H(p_A)}{H(p)}.$$

□

To simplify discussion in what follows, for a distribution $p = (p_1, \ldots, p_n)$ and a partition A of $[n]$ into groups with coarse law p_A, we use $E_T(p)$ to denote the total Shannon entropy $H(p)$, $E_B(p; A)$ to denote the between-group entropy $H(p_A)$ and $E_W(p; A)$ to denote the within-group entropy

$$E_W(p; A) := E_T(p) - E_B(p; A).$$

The specificity at level A is then given by

$$\Psi(p; A) := \frac{E_W(p; A)}{E_T(p)} = 1 - \frac{E_B(p; A)}{E_T(p)}.$$

2 Additivity

The key property of Ψ is its additivity, which makes it a natural measure of specificity across hierarchical biological structures. Suppose we have two tissues (A and B), each containing two cell types. Let the joint probability vector for a gene's expression be

$$p = (p_{\mathrm{A},1},\, p_{\mathrm{A},2},\, p_{\mathrm{B},1},\, p_{\mathrm{B},2}).$$

Summing within tissues gives $p_{\text{tissue}} = (p_{\mathrm{A}},\, p_{\mathrm{B}})$, where $p_{\mathrm{A}} = p_{\mathrm{A},1} + p_{\mathrm{A},2}$ and $p_{\mathrm{B}} = p_{\mathrm{B},1} + p_{\mathrm{B},2}$. Conditional distributions within tissues define $q_{\text{cell}|\mathrm{A}} = \big(p_{\mathrm{A},1}/p_{\mathrm{A}},\, p_{\mathrm{A},2}/p_{\mathrm{A}}\big)$ and $q_{\text{cell}|\mathrm{B}} = \big(p_{\mathrm{B},1}/p_{\mathrm{B}},\, p_{\mathrm{B},2}/p_{\mathrm{B}}\big)$.

Using the entropy representation of Ψ from Theorem 2 and the Shannon chain rule,

$$\Psi_{\text{joint}} = \Psi(p_{\text{tissue}}) + \big(1 - \Psi(p_{\text{tissue}})\big)\, \mathbb{E}_{\text{tissue}}\big[\Psi(q_{\text{cell}|\text{tissue}})\big], \tag{1}$$

where the expectation is taken over tissues with weights p_t.

Example.

Example. Consider the gene count table:

	cell 1	cell 2	total (tissue)
A	40	10	50
B	25	15	40
Total			90

so that

$$p = \left(\tfrac{40}{90}, \tfrac{10}{90}, \tfrac{25}{90}, \tfrac{15}{90}\right), \qquad p_{\text{tissue}} = \left(\tfrac{50}{90}, \tfrac{40}{90}\right).$$

The conditional distributions within tissues are

$$q_{\text{cell}|\mathrm{A}} = (0.8,\, 0.2), \qquad q_{\text{cell}|\mathrm{B}} = (0.625,\, 0.375).$$

Using base-2 Shannon entropy,

$$H(p) = -\sum_i p_i \log_2 p_i,$$

we obtain

$$H(p) = 1.8163, \qquad H(p_{\text{tissue}}) = 0.9911.$$

The within-tissue entropy is

$$H_{\text{within}} := \tfrac{50}{90}\, H(q_{\text{cell}|\text{A}}) + \tfrac{40}{90}\, H(q_{\text{cell}|\text{B}}),$$

where

$$H(q_{\text{cell}|\text{A}}) = 0.7219, \qquad H(q_{\text{cell}|\text{B}}) = 0.9544,$$

so that

$$H_{\text{within}} = 0.8253.$$

These values satisfy the Shannon chain rule

$$H(p) = H(p_{\text{tissue}}) + H_{\text{within}} = 0.9911 + 0.8253 = 1.8163.$$

Moreover,

$$E_T(p) = H(p) = 1.8163, \quad E_B(p;\text{tissue}) = H(p_{\text{tissue}}) = 0.9911, \quad E_W(p;\text{tissue}) = H_{\text{within}} = 0.8253.$$

The specificity at the tissue level is

$$\Psi_{\text{tissue}} = \Psi(p;\text{tissue}) = \frac{E_W(p;\text{tissue})}{E_T(p)} = 1 - \frac{E_B(p;\text{tissue})}{E_T(p)} = 1 - \frac{0.9911}{1.8163} = 0.4544.$$

Equivalently, the between-tissue contribution is

$$\frac{E_B(p;\text{tissue})}{E_T(p)} = \frac{0.9911}{1.8163} = 0.5456,$$

and the two normalized components sum to one:

$$0.4544 + 0.5456 = 1.$$

Thus, Ψ decomposes total specificity at this level into exactly complementary within-tissue and between-tissue contributions, with no dependence on arbitrary normalization.

3 Order invariance and hierarchical decomposition

Because Ψ is defined in terms of entropy ratios, it inherits the permutation symmetry and associative property of Shannon entropy. In particular, the value of Ψ does not depend on the order in which hierarchical partitions are applied: for example, whether one decomposes first by strain and then by tissue and sex, or by tissue and then by strain and sex, the resulting Ψ is identical.

To illustrate, consider the 8-cube dataset, in which each observation can be categorized by three binary variables: strain (A/B), tissue (brain/liver), and sex (M/F). Let p_{ijk} denote the normalized expression proportion in cell (i, j, k). Using the entropy representation of Ψ and applying the Shannon chain rule recursively,

$$\begin{aligned} \Psi_{\text{strain,tissue,sex}} &= \Psi_{\text{strain}} + (1 - \Psi_{\text{strain}})\, \mathbb{E}_{\text{strain}}\Big[\Psi_{\text{tissue}|\text{strain}} \\ &\qquad + (1 - \Psi_{\text{tissue}|\text{strain}})\, \mathbb{E}_{\text{tissue, strain}}\big[\Psi_{\text{sex}|\text{tissue,strain}}\big]\Big]. \end{aligned} \tag{2}$$

Reversing the order of marginalization yields exactly the same value, because the additive composition of Ψ mirrors the associative chain rule of entropy:

$$\begin{aligned} H(X,Y,Z) &= H(X) + H(Y \mid X) + H(Z \mid X,Y) \\ &= H(Y) + H(Z \mid Y) + H(X \mid Y,Z). \end{aligned}$$

Since

$$\begin{aligned} \Psi(X,Y,Z) &= \Psi(X) + (1-\Psi(X))\,\Psi(Y \mid X) \\ &\quad + (1-\Psi(X))(1-\Psi(Y \mid X))\,\Psi(Z \mid X,Y), \end{aligned}$$

and likewise

$$\begin{aligned} \Psi(X,Y,Z) &= \Psi(Y) + (1-\Psi(Y))\,\Psi(Z \mid Y) \\ &\quad + (1-\Psi(Y))(1-\Psi(Z \mid Y))\,\Psi(X \mid Y,Z), \end{aligned}$$

the two decompositions are algebraically equivalent. Each term corresponds to a different expansion of the same normalized entropy reduction, and their equality follows from the associativity of the Shannon chain rule.

Therefore Ψ defines a consistent, hierarchical measure of gene specificity that is independent of the order in which biological factors are considered.

References

[1] Dmitrii Konstantinovich Faddeev. On the concept of entropy of a finite probabilistic scheme. *Uspekhi Matematicheskikh Nauk*, 11(1):227–231, 1956.
